# The effect of minimizing central line days for very low birth weight infants through quality improvement

**DOI:** 10.1038/s41598-024-53163-4

**Published:** 2024-02-15

**Authors:** Jeongmin Shin, Hyun Mi Kang, Sae Yun Kim, Young-Ah Youn, Chang Won Choi, Yun Sil Chang

**Affiliations:** 1 Department of Pediatrics, Seoul St. Mary’s Hospital, College of Medicine, The Catholic University of Korea, 222 Banpo-daero, Seocho-gu, Seoul, 06591 Republic of Korea; 2 Department of Pediatrics, Seoul National University Bundang Hospital, 82 Gumi-ro 173 Beon-gil, Bundang-gu, Seongnam-si, Gyeonggi-do 13620 Republic of Korea; 3 Department of Pediatrics, Samsung Medical Center, Sungkyunkwan University School of Medicine, 81 Irwon-ro, Gangnam-gu, Seoul, 06351 Republic of Korea

**Keywords:** Paediatrics, Prognosis, Quality of life, Health care, Medical research, Risk factors

## Abstract

Blood culture proven sepsis is associated with increased mortality and morbidity. Given the extended hospitalization of very preterm infants, catheter-related blood stream infections (CRBSIs) play a substantial role in sepsis. The reported incidence of CRBSIs in neonates varies from 3.2 to 21.8 CRBSIs per 1000 catheter line days. Moreover, discrepancies in neonatal practices and potential neglect may lead to the unwarranted prolongation of central lines. This study aims to compare two distinct periods (Pre-QI vs. Post-QI) in relation to the central line insertion rate and duration, as well as blood culture proven sepsis, duration of total parenteral nutrition (TPN), and the progression of feeding. These factors are known to be associated with prolonged hospitalization and increased morbidities. A total of 210 very low birth weight infants (VLBWIs), defined as either less than 32 weeks of gestational age (GA) or weighing less than 1500 g, were admitted to the Neonatal Intensive Care Unit (NICU) at Seoul St. Mary’s Hospital, The Catholic University of Korea, between January 2020 and June 2023. Fourteen infants were excluded from the study as they did not survive beyond 1 month of life, and one was excluded due to a congenital anomaly. Consequently, the analysis included 195 VLBWIs. The Quality Improvement (QI) initiative began in January 2022, marking the division into two distinct epochs: the Pre-QI period, encompassing the years 2020 to 2021, and the Post-QI period, spanning from 2022 to 2023. The primary outcome measures included PICC insertion rates, duration, and feeding advancement or feeding-related complications. The hospital outcome measures were also compared between the two periods. A total of 195 VLBWI were included in the analysis. The birth weight was significantly lower in the pre-QI period, with an average of 1023 g compared to 1218 g (P < 0.001). Severe BPD ≥ moderate was significantly lower in the post-QI period (36.2% vs. 53.9%) (P < 0.001) along with shorter mechanical ventilation days (12 ± 29 vs. 22 ± 27) (P = 0.046). The PICC insertion rate was significantly decreased from 95.6% in pre-QI period compared to 55.2% in post-QI period (P < 0.001) along with a notable reduction in blood culture-proven sepsis (25.6% vs. 10.5%, P = 0.008). CRBSI rate was reduced from 1.3 to 1.1 per 1000 catheter days in the post-QI period. Moreover, the time required to achieve full enteral feeding of 100 mL/kg/day was significantly shorter in the post-QI (24 ± 23 vs. 33 ± 25) (P = 0.006). Multivariable logistic regression analysis for sepsis revealed that both birth weight and pre/post QI consistently demonstrated an association with lower sepsis rates in the Post-QI period. QI has the potential to reduce the burden of unnecessary interventions and blood culture proven sepsis rate along with CRBSI rate, thereby, optimizing the better care of very preterm babies.

## Introduction

Vascular access is an essential component of care in the neonatal intensive care unit (NICU) for nutritional support and medication administration. While the umbilical vein catheter is usually employed during the initial 3 days, peripherally inserted central catheters (PICCs) are the preferred mode of central venous access once umbilical venous catheters are either discontinued or cannot be inserted. It is important to note that prolonged use of umbilical vein catheters beyond 10 days is discouraged due to an increased risk of sepsis or catheter-related blood stream infections (CRBSIs). However, without daily check up for central line necessity, unnecessary placement may prolong the peripherally inserted central line days which may also increase sepsis or CRBSIs. The Centers for Disease Control and Prevention (CDC) reported that adoption and implementation of evidence-based practices, such as bundled central line interventions, have significantly decreased 44% national CLABSI rates between 2008 and 2012^1^. Concerted team efforts such as bundled central line interventions in the NICU population led to reductions in sepsis rates in very preterm birth weight infants (VLBWI) who have a high risk of infection due to biological immaturity, frequent invasive procedures and prolonged requirement for respiratory support and parenteral nutrition^2,3^. Sepsis in NICU is a major cause of morbidity and mortality for VLBWI because prematurity is a consistent independent risk factor for neonatal sepsis; up to 20% of mortalities in VLBWI are caused by sepsis, and infants with sepsis are at a nearly three-fold risk of mortality than those without sepsis^4,5^. Even more, extremely premature neonates may have a greater risk of infection due to immunocompromised immunity including skin, frequent invasive procedures and prolonged requirement for respiratory support, parenteral nutrition and prolonged hospitalization^6,7^. Further, they are at increased risk for complications and death if they develop an infection because the symptoms are nonspecific, and overlap with noninfectious diseases such as lung or abdominal conditions. As a long term outcomes, these high risk infants with sepsis manifested adverse neurodevelopmental outcomes and growth impairment as compared to those of uninfected infants^8^.

As a result, inspired by successful quality improvement initiatives related to central line bundles that have been reported to reduce sepsis in the NICU, we initiated a QI project aimed at minimizing PICC duration and avoiding routine insertion of PICC for VLBWI. The goal was to potentially avoid PICC insertion for VLBWI born at < 32 weeks of gestational age (GA) or weighing less than 1500 g, as long as they were stable enough to progress with feeding without the need for a PICC.This study aims to assess whether the QI collaborative successfully reduced PICC duration and, potentially, prevented the need for PICC insertion, leading to improved clinical outcomes for VLBWI in the post-QI period.

## Methods

### Study design

**Figure 1:**
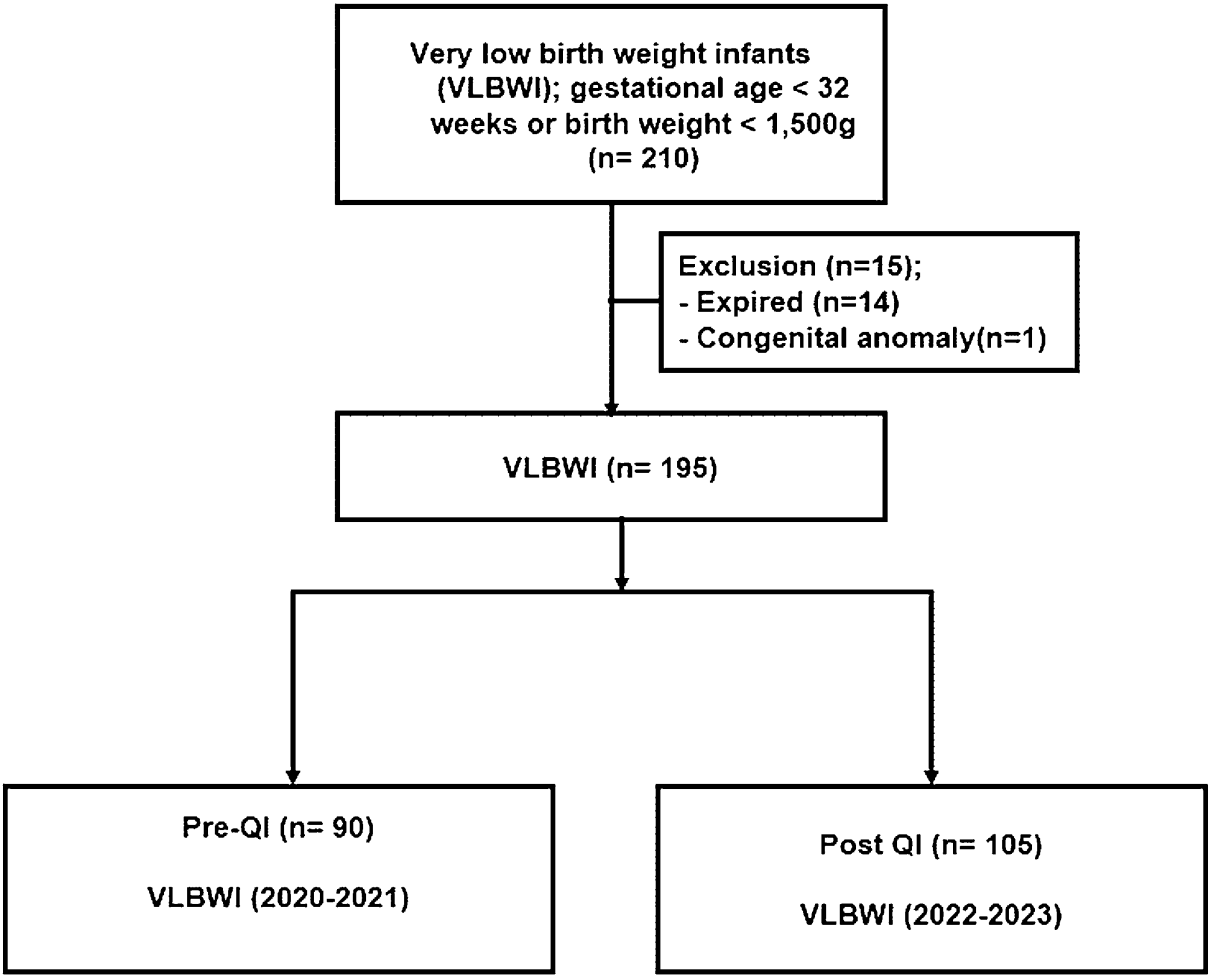
Study flow chart. *VLBWI* very low birth weight infants, *QI* quality improvement.

The initiative to minimize central line days through a quality improvement (QI) project was prospectively implemented in December 2021 for high risked VLBWIs, born either less than 32 weeks of gestational age (GA) or weighing less than 1500 g between January 2020 and June 2023. A total of 210 very low birth weight infants (VLBWIs) were born during this study period; 15 VLBWI were excluded from this study for 14 infants died within a month of life and one being diagnosed with a congenital anomaly. As a result, a total of 195 VLBWIs were enrolled for the analysis (Fig. 1). Before QI project to reduce sepsis, the active practice of diagnosing CRBSIs was not consistently implemented and routine PICC insertion was initiated even hemodynamically stable VLBWI and PICC insertion was kept longer due to concerns about possibility of unstable conditions, as perceived by clinicians. After QI, we assessed the PICC need daily and tried to remove PICC insertion for VLBWI who were hemodynamically stable.

### Data definitions

The PICC insertion period was defined as the duration from the initial insertion date to the date of final removal. In instances where both removal and reinsertion occurred on the same day for the purpose of changing to a new insertion, it was categorized as a replacement rather than a separate removal and insertion. The PICC was counted as central line from day 1 starting on the insertion day. We did not encounter any cases with two PICCs, but rather with additional peripheral lines for further medication. Sepsis was diagnosed in a patient with symptoms and/or signs of systemic infection. Blood proven sepsis was defined if a patient had a positive result for one or more bacterial or fungal cultures obtained from blood of the infants with clinical signs of infection or treated with antibiotics for 5 or more days or treated for a shorter period if the patient died^9,10^. All instances of sepsis in this study were classified as late-onset sepsis (LOS), as they occurred after 10 days of life, at least 3 days after the insertion of the PICC insertion date. Catheter-related blood stream infection (CRBSI) was diagnosed based on (1) a positive catheter tip culture or a positive blood culture drawn from the central venous catheter, (2) clinical symptoms and/or signs of systemic infection, and (3) a positive peripheral blood culture. The CRBSI rate was calculated by episodes per 1000 catheter days. It is further defined as a laboratory-confirmed bloodstream infection not related to an infection at another site that develops within 48 h of central line placement. The minimum duration for which a central line must have been in place for CRBSI was more than 3 days in our study. Bronchopulmonary dysplasia (BPD) was diagnosed if oxygen use exceeding 0.21% was still needed at a corrected gestational age of 36 weeks. Necrotizing enterocolitis (NEC) was defined as grade II or higher using Bell’s classification. IVH ≥ 3 was defined as active bleeding with enlarged ventricles, and the grade designation was based on Drs. Papile and Levene’s classification criteria^11^. Full feeding was defined as the attainment of enteral feeding exceeding > 100 mL/kg/day, at which point the PICC could be removed.

### Data collection

Point of care data were entered into a Korean neonatal network (KNN) QI database by clinicians for all VLBWI who had PICC insertion. Korean neonatal network was established by the Korean Society of Neonatology and Korea Centers for Disease Control and Prevention in 2013. This database prospectively registered all of the clinical information of VLBWIs admitted to the 89 voluntarily participating neonatal intensive care units (NICUs), covering greater than 80% of very preterm infants in South Korea^9^. The study was approved by the Ethics Committee of Seoul St. Mary’s Hospital, The Catholic University of Korea, South Korea (IRB# KC13ONMI0228). The requirement for informed consent from the study subjects was waived by the IRB of by the Ethics Committee of Seoul St. Mary’s Hospital due to the retrospective study design. All methods were conducted in accordance with applicable guidelines and regulations.

### Statistical analysis

Statistical Process Control (SPC) allows to identify situations that require special attention and potential adjustments to standard SPC charts. SPC methods were developed to facilitate the analysis of time-series data, enabling the observation of general patterns and the effectiveness of QI study. We recorded the duration of PICC for each infant in an excel spreadsheet and created a SPC curve, with the x-axis representing the monthly timeline.

Continuous variables are expressed as means ± standard deviation (SD) and categorical variables are expressed as numbers and proportions. Pearson’s χ^2^ test was used to analyze categorical variables between two groups. Student’s *t* test was used to compare continuous variables between groups. Statistical analysis was conducted using SPSS software, version 26.0 (IBM, Corp., Armonk., NY, USA). A *P*-value of < 0.05 was considered statistically significance.

## Results

A total of 210 VLBWI were born were between January 2020 and June 2023. Among these, the 14 VLBWI who expired and one with a chromosomal anomaly were not eligible for this study and were consequently excluded from the analysis. Therefore, a total of 195 VLBWI were included in the analysis to assess the impact of the QI initiative that commenced in January 2022 (Fig. 1).

### Demographics and baseline characteristics of study population

The average gestational age of VLBWI in the pre-QI activity period (those born between January 2020 to November 2021) was 28 weeks and 4 days, whereas the post-QI group had a mean gestational age of 29 weeks and 1 day, with an average weight of 1218 g. The birth weight was significantly lower in the pre-QI period, with an average of 1023 g (P < 0.001). The rates of severe bronchopulmonary dysplasia (BPD) ≥ moderate was significantly lower in the post-QI period (36.2% vs. 53.9%) (P < 0.001) along with shorter mechanical ventilation days (12 ± 29 vs. 22 ± 27) (P = 0.046). Additionally, patent ductus arteriosus (PDA) ligation rate (5.7% vs. 18.9%) was also significantly lower in the post-QI period. However, the other clinical outcomes did not differ significantly between the two periods (Table 1).

**Table 1:** Clinical characteristics and outcomes of VLBWIs (n = 195).

	Pre QI (n = 90)	Post QI (n = 105)	*P*-value
Gestational age, weeks	28^+4^ ± 2^+5^	29^+1^ ± 2^+4^	0.208
Birth weight, g	1023.5 ± 270.4	1218.0 ± 434.9	< 0.001
Male, n (%)	50 (50.5%)	53 (48.2%)	0.782
Antenatal steroid use	67 (78.8%)	82 (78.8%)	1.000
Maternal chorioamnionitis	29 (34.1%)	20 (19.2%)	0.030
Resuscitation at delivery*	90 (1000%)	101 (96.2%)	0.126
RDS	72 (80%)	75 (71.4%)	0.185
Pneumothorax	4 (4.4%)	6 (5.7%)	0.755
Pulmonary hemorrhage	4 (4.4%)	8 (7.6%)	0.390
Neonatal seizure	8 (8.9%)	3 (2.9%)	0.116
PDA required treatment^‡^	30 (33.3%)	28 (26.7%)	0.347
PDA ligation^‡^	17 (18.9%)	6 (5.7%)	0.007
ROP operation	5 (5.6%)	11 (10.6%)	0.296
BPD ≥ moderate^‡^	48 (53.9%)	38 (36.2%)	0.014
Invasive mechanical ventilation, days^‡^	22 ± 27	12 ± 29	0.046
IVH ≥ 3^‡^	6 (6.7%)	6 (5.7%)	1.000
Hospitalization, days^‡^	96 ± 44	88 ± 45	0.733

*Resuscitation included oxygen use or positive pressure ventilation or intubation.

^‡^Patients who died were excluded.

*VLBWI* very low birth weight infants, *g* gram body weight, *RDS* respiratory distress syndrome, *PDA* patent ductus arteriosus, *ROP* retinopathy of prematurity, *BPD* bronchopulmonary dysplasia, *IVH* intraventricular hemorrhage.

### QI activity related outcomes

The PICC insertion rate was significantly decreased from 95.6% in pre-QI period compared to 55.2% in post-QI period (P < 0.001) along with a notable reduction in blood culture-proven sepsis (25.6% vs. 10.5%, P = 0.008). Moreover, the time required to achieve full enteral feeding of 100 mL/kg/day was significantly shorter in the post-QI (24 ± 23 vs. 33 ± 25) (P = 0.006). However, no significant differences were observed in the incidence of NEC ≥ stage 3 (3.3% vs. 0.0%, P = 0.059) or PICC duration between the two periods. However, the CRBSI rate was reduced to 1.1/1000 catheter days during the post-QI period compared to the pre-QI period of CRBSI rate of 1.3/1000 catheter days (P = 0.045) (Table 2).

**Table 2:** Quality improvement related outcomes of VLBWIs (n = 195).

	Pre-QI (n = 90)	Post-QI (n = 105)	*P*-value
PICC insertion rate	86(95.6%)	58(55.2%)	< 0.001
PICC duration, days	34 (± 22)	34 (± 23)	0.740
Sepsis, blood culture proven	23 (25.6%)	11 (10.5%)	0.008
CRBSI episodes	4 (4.4%)	2 (3.4%)	1.000
CRBSI/1000 catheter days	1.3	1.1	0.045
NEC ≥ stage 2	3 (3.3%)	0 (0.0%)	0.097
Full enteral feeding reached (100 mL/kg/day)	33 (± 25)	24 (± 23)	0.006
TPN duration^‡^	36 (± 27)	30 (± 31)	0.177

^‡^Patients who died were excluded.

*VLBWI* very low birth weight infants, *PICC* peripherally inserted central catheter, *CRBSI* catheter- related blood stream infections,  *NEC* necrotizing enterocolitis, *TPN* total parental nutrition.

### Those who did not insert PICC according to gestational age

Minimizing the duration of inserted PICC is crucial, but opting for unnecessary PICC placement may be a better choice for VLBWI who were stable enough to progress to feed with a G-tube and eventually oral feeding. Notably, in the Pre-QI group, all VLBWI, regardless of gestational age, had PICCs inserted. However, in the Post-QI group, 20 infants born at 31 weeks of gestational age demonstrated tolerance without requiring PICC insertion. Additionally, no PICCs were inserted for 4 infants born at 30 weeks and 7 infants born at 29 weeks. Remarkably, survival to discharge without PICC insertion was observed for 2 infants born at 26 weeks and 3 infants born at 27 weeks of gestational age. Conversely, those born at 23 weeks and 24 weeks all necessitated PICC insertion (Table 3).

**Table 3:** The VLBWI who did not have PICC insertion during NICU hospitalization according to gestational age.

Gestational age (weeks)	Pre-QI (n = 4)	Post-QI (n = 49)
24	0	0
25	0	0
26	0	2
27	0	3
28	0	1
29	0	7
30	0	4
31	0	20

*VLBWI* very low birth weight infants, *PICC* peripherally inserted central catheter, *NICU * neonatal intensive care unit.

### Sepsis risk factor in a multivariable logistic regression analysis

Sepsis risk factors were examined through a multivariable logistic regression analysis. Given the significance of birth weight as a contributing factor to sepsis, we further investigated the impact of potential confounding factors on sepsis in VLBWI. Our analysis included birth weight, pre/post QI, and severe BPD, all of which were identified as significant risk factors for sepsis. The results of the analysis revealed that both birth weight and pre/post QI consistently demonstrated an association with lower sepsis rates in the Post-QI period (Table 4).

**Table 4:** Univariate and multivariate logistic regression analysis for sepsis.

	Univariate		Multivariate	
	P	Odds ratio	P	Odds ratio
Birth weight, g	< 0.001	0.998	0.009	0.998
QI pre/post	0.007	0.341	0.047	0.437
Severe BPD	0.016	2.585	0.955	0.971
PDA ligation	0.087	2.350		

*Multivariate analysis: adjusted for birth weight, QI pre/post, severe BPD.

Variables included in the multivariable logistic regression model: birth weight, QI pre/post and BPD.

*QI* quality improvement, *BPD* bronchopulmonary dysplasia, *PDA* patent ductus arteriosus.

**Figure 2:**
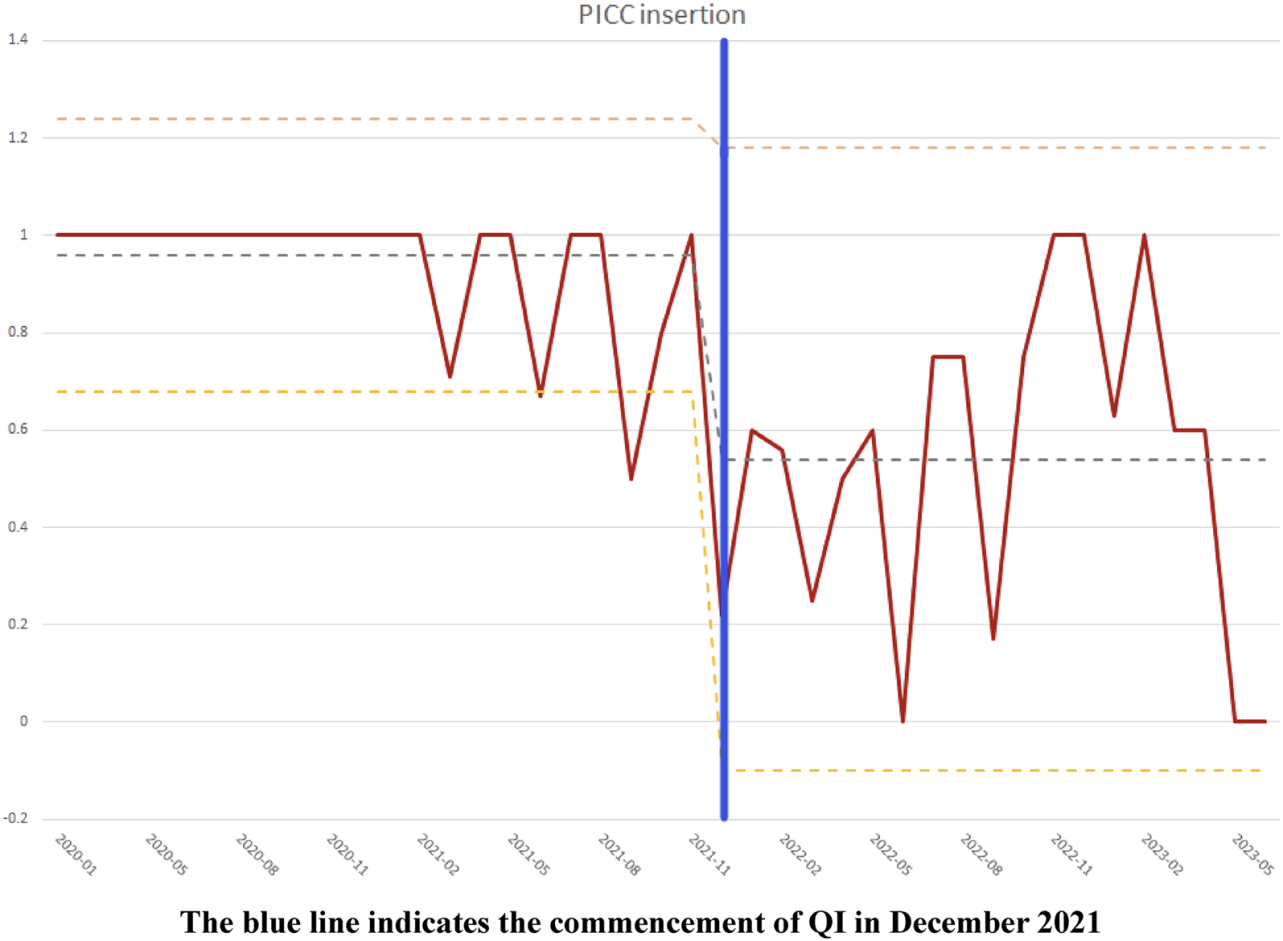
Statistical process control chart for PICC (peripherally inserted central catheter) insertion duration; Pre QI vs. Post QI.

The Statistical Process Control; Pre QI vs. Post QI showed how QI collaborative started on December 2021 changed a downward curve for PICC insertion over time with data plotted in time order which enables process QI performance monitoring. The vertical axis has 1.0 as the center line, with 1.5 as the upper control limit and 0.4 as the lower control limit. The SPC chart indicates special cause variations when there are 14 or more consecutive decreasing or increasing points. In our SPC chart for PICC duration (Pre-QI vs. Post-QI), several downward points appeared right after the QI initiative. However, these points subsequently shifted upward, implying a longer PICC duration comparable to the period before QI. This shift may suggest an increase in very preterm babies requiring the PICC without achieving full feeding (Fig. 2).

### Ethics approval

For this retrospective study, a formal consent was waived due to chart review in this study; any personal data was protected. The ethical approval was obtained from the Catholic University of Korea, Seoul St. Mary’s Hospital, Institutional Review Board.

### Consent to participate

We, each author listed on the manuscript, have seen and approved the submission of this version of the manuscript and take full responsibility for the manuscript. We have no conflicts of interest to declare.

## Discussion

In our study, The PICC insertion rate was significantly decreased from 95.6% in pre-QI period compared to 55.2% in post-QI period (P < 0.001) along with a notable reduction in blood culture-proven sepsis (25.6% vs. 10.5%, P = 0.008). CRBSI rate was reduced from 1.3 to 1.1 per 1000 catheter days in the post-QI period. Further, a multivariable logistic regression analysis for sepsis revealed that both birth weight and pre/post QI consistently demonstrated an association with lower sepsis rates in the Post-QI period. The use of PICCs in NICUs has significantly increased in recent years^1–3,5–7^. This rising usage has drawn attention to the associated complications, mostly thrombosis and infection. However, many PICC-related complications encompass infections, clot formation, malposition and malfunction/occlusion^12–15^. Additionally, rare but severe complications such as extravasation into the pleural, pericardial, or peritoneal space, arrhythmia and line fracture with embolization have been reported^15,16^.

Being aware of these serious complications, some PICCs may be inserted without clinically valid reasons. In our unit, the practice involves removing the umbilical vein catheter (UVC) by day 3 of life due to possible malposition or abdominal distension, but after all to reduced unwanted sepsis. Butler-O’Hara et al.^15^ also reported that UVCs left in situ more than 7 days were more likely to be associated with CLABSIs than PICCs. Eventually, replacing UVC with a PICC for total parenteral nutrition (TPN) and medications after 3 days of life was our routine practice. Before QI initiative, line removal was tried when the feeding volumes from a wide range of 100 to 150 mL/kg per day were achieved. In our post QI period, our team agreed to standardize this practice and narrowed a recommendation for line removal at enteral feedings of 100 mL/kg per day. We daily assessed the need for a central line with the question “Do we really need the line today?” Before the QI initiative, PICC insertion was kept longer due to concerns about possibility of unstable conditions, as perceived by clinicians.

In our study, the QI initiative successfully reduced PICC insertion rates compared to the pre-QI period. It also showed enhanced outcomes related to PICC, such as a reduced incidence of blood-culture proven sepsis and earlier achievement of full enteral feeding in the NICU. Further, a multivariable logistic regression analysis for sepsis revealed that both birth weight and pre/post QI consistently demonstrated an association with lower sepsis rates in the Post-QI period. PICC insertion was routine due to concerns about possibility of unstable conditions, as perceived by clinicians. The CRBSI rate at our NICU during the pre-QI period was 1.3 per 1000 catheter days, which was already lower than the 3.2–21.8 CRBSI rates reported in other studies prior to any interventions^13–15^. One of the main interventions administered in this study was minimizing the maintenance of PICC days and possibly avoiding the insertion of PICC if the VLBWI who were born at > 30 weeks of GA. However, prior to this intervention, our NICU had already undergone interventions including routine environmental surveillance, hand washing education, and changing environmental surfaces of high-touch areas to easily disinfectable surfaces, which reduced the CRBSI rate from 3.7 to 2.0 per 1000 catheter days^16^. In this study, by further incorporating protocols to minimized the use of PICCs, the QI initiative successfully reduced PICC insertion rates compared to the pre-QI period, resulting in decreased CRBSI rates per 1000 catheter days, as well as a lower rate of blood-culture proven sepsis and earlier attainment of full enteral feeding during NICU.

The overall compliance rates for reducing PICC maintenance showed improving trends in the SPC chart right after QI. The SPC chart indicates special cause variations when there are 14 or more consecutive decreasing or increasing points. In our SPC chart for PICC duration (Pre-QI vs. Post-QI), several downward points appeared right after the QI initiative. However, these points subsequently shifted upward, implying a longer PICC duration comparable to the period before QI. This shift may be explained by increased number of very preterm babies born around the time requiring the PICC insertion longer without achieving full feeding. One limitation in establishing significance was this study was rather short for 15-month observation period. Based on the trend line and significant reduction in blood culture proven sepsis, a longer period of observation might have revealed a significant relationship between the QI collaborative and PICC duration and CLABSI reduction.

Further, after the QI initiative in December 2021, in the Post-QI group, 20 infants born at 31 weeks of gestational age demonstrated tolerance without requiring PICC insertion. Additionally, no PICCs were inserted for 4 infants born at 30 weeks and 7 infants born at 29 weeks. Remarkably, survival to discharge without PICC insertion was observed for 2 infants born at 26 weeks and 3 infants born at 27 weeks of gestational age (Table 3) and remained hemodynamically stable and achieved nutritional goals by 10–14 days of age. Prior to the QI project, routine PICC insertion was initiated even for hemodynamically stable VLBWI, and PICC insertion was prolonged due to concerns about potential unstable conditions, as perceived by clinicians. After the QI project, it became evident that not all VLBWI required PICC insertion, and the practice of routinely inserting PICCs out of clinicians' fear should be avoided in the future.

It was possible to execute a successful NICU collaborative in minimizing central line days and reducing blood culture proven sepsis due to ongoing QI efforts. The QI initiative highlighted the importance of conducting objective assessments of infants’ conditions after Umbilical Venous Catheter (UVC) removals, with a focus on minimizing the need for subsequent PICC insertion. In cases where PICC insertion is judged to be necessary, it is imperative to minimize its duration through regular daily assessments and the use of a daily checklist. Therefore, establishing more specific criteria for the appropriate indications, maintenance, and care of PICCs is essential to ensure patient safety.

There were several limitations to this study. Firstly, the overall number of study participants was relatively small; however, it remains significant as only VLBWIs were included. Secondly, the number of CRBSI episodes during post-QI periods was relatively low with decreased sepsis rate. Nevertheless, the QI initiatives were successful in reducing sepsis and CRBSI rates.

## Conclusion

The QI project has proven beneficial in significantly reducing PICC insertion rates, particularly those born at < 30 weeks of gestational age (GA). Additionally, there was a significant reduction of blood culture proven sepsis. However, longer QI collaborative evaluation is needed to assess the effects of this project on PICC duration rate, CLABSI and other clinical outcomes. Further studies on the long-term effects of this QI are warranted.

## Data Availability

The datasets used during the current study are available from the corresponding author upon request. Medical records are available in the Archive of the Department of Pediatrics of the Seoul St. Mary’s Hospital.
